# HIV Preventive Measures’ Adoption Among Men Who Have Sex With Men in West and Central Africa: A Scoping Review On Pre-exposure Prophylaxis and Condom Use

**DOI:** 10.1007/s10900-026-01600-y

**Published:** 2026-08-10

**Authors:** Jolayemi Afolabi, Sally Arrey, Ak Narayan Poudel

**Affiliations:** School of Human and Health Sciences, University of Huddersfield, Queensgate, Huddersfield UK

**Keywords:** HIV prevention, Men who have sex with men (MSM), Pre-exposure prophylaxis (PrEP), Condom use, West and Central Africa

## Abstract

Pre-exposure prophylaxis (PrEP) and condom use have contributed to a global decline in the incidence of human immunodeficiency virus (HIV) infections. However, despite concerted preventive efforts, HIV infections remain highly prevalent in West and Central Africa (WCA) among key populations, particularly among men who have sex with men (MSM). We conducted a scoping review to gain a better understanding of the status of PrEP adoption and condom use as effective HIV prevention measures among MSM in WCA and identified gaps. The review was conducted based on JBI Scoping Review Guidelines spanning 1 January 2017 to 10 April 2025. A comprehensive search was conducted in PubMed, CINAHL Ultimate, PsycINFO, ProQuest, Scopus, and OpenAIRE. This was supplemented with a manual search of reference lists, websites, and other online sources to capture key information relevant to the review objectives. Emerging patterns from the data were described and synthesised. Of the 713 identified studies, 22 studies comprising 19 quantitative and 3 qualitative studies were included in the final synthesis. The results indicate uneven with generally low to high uptake of PrEP, persistent inconsistencies in condom use, and risk compensation behaviours among MSM in WCA. Given that our findings are primarily from quantitative studies, additional qualitative studies on the lived experiences of MSM regarding these preventive measures are needed. From a policy implementation perspective, emphasis should be placed on MSM-inclusive, context-sensitive, and community-aligned HIV prevention strategies in WCA.

## Background

The global HIV situation remains alarming. In 2023, an estimated 39.9 million people were living with HIV, of whom 1.3 million were new cases [1]. There were 630,000 AIDS-related deaths [1]. Sub-Saharan Africa (SSA) accounts for over two-thirds of the global HIV infections [2, 3]. HIV prevention strategies include consistent condom use and pre-exposure prophylaxis (PrEP), a more recent, innovative biomedical strategy [3]. PrEP is a highly effective, evidence-based oral antiretroviral medication recommended for use in individuals at high risk of contracting HIV, such as key populations, which has the potential to greatly impact the HIV epidemic globally [4, 5]. Key populations (KPs) comprise individuals at high risk of contracting HIV/AIDS because they engage in activities such as unprotected sex, penile-anal sex, multiple sexual partners, and substance use [6–8]. In the context of HIV, KPs encompass sex workers, gay men and other men who have sex with men (MSM), people who inject drugs, and those who identify as transgender [1]. KPs are prevalent across Africa and globally, and PrEP serves as an additional HIV preventive strategy for this group [6–9].

The HIV epidemic in West and Central Africa (WCA) disproportionately affects KP groups, particularly MSM, who accounted for 69% of all new HIV infections in 2019, according to UNAIDS [9]. The 7.1% HIV prevalence among MSM far exceeds the 1.3% rate in the general population in WCA [10]; this is in contrast to a prevalence of 12.9% among MSM in Eastern and Southern Africa (ESA), where the general population prevalence is 5.9% [11]. MSM accounted for 18% of new HIV infections in WCA, as opposed to only 3% in ESA [10]. This huge disparity in HIV prevalence between WCA and ESA necessitates a better understanding of the status of HIV prevention strategies and highlights the need for region-specific, MSM-centric interventions.

Despite the significant impact of KPs on the HIV burden, their engagement with HIV prevention and treatment services is critically suboptimal due to socio-legal impediments, such as homophobia, stigmatisation, discrimination, violence, and penalisation of homosexual acts [12–15]. The high rate of HIV transmission among MSM continues to pose a substantial challenge globally, despite efforts to reduce incidence and the overarching goal to halt the HIV epidemic [16]. Potential facilitators of HIV transmission among KPs include the elevated transmission probability associated with every receptive anal intercourse, a high prevalence of sexually transmitted infections, frequent condomless anal sex, high numbers of male sexual partners, and participation in extensive sexual interconnections [17].

To stem the tide of the global HIV/AIDS crisis and eradicate AIDS by 2030, the United Nations General Assembly aims to achieve the 95–95–95 targets [11, 18]. The 2025 UNAIDS 95-95−95 targets imply that 95% of individuals who are living with HIV should know their HIV status; 95% of people within the subpopulation who are living with HIV and who know their HIV status are on antiretroviral treatment (ART); 95% of those on ART have suppressed viral loads [19]. These targets work best when they are combined with preventive strategies as part of a multifaceted approach, of which PrEP is a critical one [19].

Given the significantly higher HIV prevalence among MSM in WCA relative to the general population, and in comparison, with ESA [10, 11], the status of adoption of preventive measures remains unclear despite their implementation. We need a better understanding of the status of PrEP adoption and condom use among MSM. This is crucial for revising the current HIV prevention policy and program implementation across WCA. In response to this need, a scoping review was undertaken with the aim of systematically mapping existing literature for available evidence on PrEP adoption and condom use among MSM in WCA and to define the research gap. Accordingly, the objectives of this review were to explore the available evidence regarding the adoption of PrEP and condom use among MSM in WCA; to examine the geographic variations of prevalence and patterns of use; and to identify behavioural shifts following PrEP initiation that may reflect risk compensation. In addition, the review investigated the use of PrEP and condoms singly or in combination. This scoping review documented the status of PrEP uptake and condom use among MSM in WCA.

## Methods

This scoping review was performed using the JBI methodological guidance for the conduct of scoping reviews [20]. This comprised defining the research question in alignment with the objective(s), describing the review approach, charting key information, presenting and analysing the results, and discussing the implications. The review conformed to the Preferred Reporting Items for Systematic Reviews and Meta-analysis Protocols for Scoping Reviews (PRISMA-ScR) [21]. A completed PRISMA-ScR checklist, detailing how each item was addressed in the conduct and reporting of this scoping review, is provided immediately after the reference list.

The review protocol was registered in the Open Science Framework (OSF) repository and is accessible via the following link- https://osf.io/26tcv/overview?view_only=6e7b22834b3c46ed8609e87ed98479fa.

### Defining the Research Question

The research question “What is the status of PrEP adoption and condom use as HIV prevention measures among MSM in WCA?” was developed using the Population, Concept, and Context (PCC) framework where P was defined as MSM (including gay and bisexual men), first C as the adoption of Oral PrEP and condom use as HIV prevention measures, and second C as WCA [22]. For this review, adoption refers to the routine incorporation of a prevention measure into regular practice or behaviour.

### Eligibility Criteria

We included studies in English providing data specifically on MSM, including gay men and bisexual men aged 18 and above, within the context of PrEP uptake and or condom use in WCA. Countries in WCA include Benin, Burkina Faso, Cape Verde, Gambia, Ghana, Guinea, Guinea-Bissau, Liberia, Mali, Mauritania, Niger, Nigeria, Senegal, Côte d’Ivoire, Sierra Leone, Togo, Angola, Cameroon, Central African Republic, Chad, Republic of the Congo, Democratic Republic of the Congo, Equatorial Guinea, Gabon, São Tomé and Príncipe. For studies including data from other demographics, such as female sex workers (FSWs) or people who inject drugs (PWID), only the data specific to MSM, including gay men and bisexual men, were extracted and analysed. Peer-reviewed studies and grey literature in English language, including cross-sectional studies, cohort studies, and qualitative research designs published from 1 January 2017 to 10 April 2025, were included (inclusion and exclusion criteria are outlined in Table 1). The commencement date of January 2017 was chosen to align with the onset of the PrEP implementation for MSM in WCA. Search terms included “PrEP,” “Pre-exposure prophylaxis”, “men who have sex with men,” “gay”, “MSM”, “homosexual men”, “Condom use”, “West Africa”, “Central Africa”, “HIV risk reduction**”** (full search terms are outlined in Table 2).

**Table 1:** Research framework and eligibility criteria

Inclusion criteria	Exclusion criteria
Studies involving MSM or gay men or bisexual men, with data for individuals aged 18 and above; articles including other population groups such as transgender women, female sex workers, or people who inject drugs, as long as they also focused on MSM, gay, or bisexual men; and provided disaggregated data for each study population, including MSM.	Studies focusing exclusively on populations other than MSM, gay, or bisexual men do not report disaggregated data for these groups or fail to specify participant age.
Articles that reported on PrEP use and or condom use, the patterns of PrEP and condom use over the years, the association between condom and PrEP use (risk compensation) and dual HIV prevention strategies (PrEP and condom) with full text availability.	Studies that focused solely on other prevention methods that lacked analysis of usage trends or their relationship (including risk compensation), did not address dual use, offered only brief mentions without substantive data, or were unavailable in full text.
Primary studies conducted in West and Central Africa, comprising 25 countries.	Studies conducted outside West and Central Africa.
All empirical study designs from both peer-reviewed and grey literature.	Studies that are non-original works, such as commentaries, editorials, blogs, news articles, reviews, duplicates, meta-analyses, systematic and scoping reviews, conference abstracts, notes, and opinion pieces.
Studies published in English language.	Studies published in languages other than English.
Studies published from 1 January 2017 to 10 April 2025.	Studies published before 2017.

## Methodological Approach to Evidence: Planning, Searching, and Selection

### Planning the Search Strategy

The selection of Medical Subject Headings (MeSH) terms was guided by the central research question of the scoping review. To enhance the comprehensiveness of the search strategy, synonymous terms for each key concept were also identified and applied across various databases, tailored to the specific search functionalities of each platform. An extensive preliminary search was conducted to ensure the effectiveness of our search strategy and to evaluate the suitability of the selected electronic databases and the keywords chosen.

### Search Strategy

Thorough searches were executed in the CINAHL Ultimate (EBSCO), ProQuest, Scopus, PsycInfo, PubMed, and in OpenAIRE, a grey literature database, to identify relevant studies for this review (Table 2). These searches covered the period from 1 January 2017 to 10 April 2025. We complemented the electronic database searches with a manual review of reference lists and relevant websites. In line with the PCC framework (Table 1), the search terms included (a) Population: “men who have sex with men”, MSM, “homosexual*”, “gay”, “bisexual*”, “sexual minority men”, “queer men”; (b) Concept: “condom use”, “condoms”, “male condom”, “PrEP”, “pre-exposure prophylaxis”, “HIV prevention”, “risk reduction”, “HIV”, “AIDS”, “human immunodeficiency virus”, “acquired immunodeficiency syndrome”; and (c) Context: “West Africa”, “Central Africa”, and all 25 constituent countries, as listed in Table 2.

**Table 2:** Databases and the search terms

Database	Search terms
Scopus	(“men who have sex with men” OR MSM OR “homosexual men” OR homosexual* OR gay OR bisexual* OR “sexual minority men”) AND (“condom use” OR condoms OR “male condom” OR PrEP OR “pre-exposure prophylaxis” OR “HIV prevention” OR “HIV risk reduction”) AND (HIV OR AIDS OR “human immunodeficiency virus” OR “acquired immunodeficiency syndrome”) AND (“West Africa” OR “Central Africa” OR Benin OR Burkina Faso OR “Cape Verde” OR “Cabo Verde” OR Gambia OR Ghana OR Guinea OR “Guinea-Bissau” OR Liberia OR Mali OR Mauritania OR Niger OR Nigeria OR Senegal OR Côte d’Ivoire OR “Sierra Leone” OR Togo OR Angola OR Cameroon OR “Central African Republic” OR Chad OR Congo OR “Republic of the Congo” OR “Democratic Republic of the Congo” OR “DR Congo” OR “Equatorial Guinea” OR Gabon OR “São Tomé and Príncipe”)
EBSCO-CINAHL Ultimate	(MH “Homosexuality, Male” OR MSM OR homosexual* OR gay OR bisexual* OR “men who have sex with men” OR “sexual minority men”) AND (MH “Condoms” OR “condom use” OR condoms OR “male condom” OR MH “Pre-Exposure Prophylaxis” OR PrEP OR “HIV prevention” OR “HIV risk reduction”) AND (MH “HIV Infections” OR HIV OR AIDS) AND (“West Africa” OR “Central Africa” OR Benin OR Burkina Faso OR “Cape Verde” OR “Cabo Verde” OR Gambia OR Ghana OR Guinea OR “Guinea-Bissau” OR Liberia OR Mali OR Mauritania OR Niger OR Nigeria OR Senegal OR Côte d’Ivoire OR “Sierra Leone” OR Togo OR Angola OR Cameroon OR “Central African Republic” OR Chad OR Congo OR “Republic of the Congo” OR “Democratic Republic of the Congo” OR “DR Congo” OR “Equatorial Guinea” OR Gabon OR “São Tomé and Príncipe”)
PubMed	(“men who have sex with men“[MeSH Terms] OR MSM OR “homosexual men” OR homosexual* OR gay OR bisexual* OR “sexual minority men”) AND (“condoms“[MeSH Terms] OR condoms OR “condom use” OR “male condom” OR “pre-exposure prophylaxis“[MeSH Terms] OR PrEP OR “HIV prevention” OR “HIV risk reduction” OR “HIV biomedical prevention”) AND (“HIV infections“[MeSH Terms] OR HIV OR AIDS OR “human immunodeficiency virus” OR “acquired immunodeficiency syndrome”) AND (“West Africa” OR “Central Africa” OR Benin OR Burkina Faso OR “Cape Verde” OR “Cabo Verde” OR Gambia OR Ghana OR Guinea OR “Guinea-Bissau” OR Liberia OR Mali OR Mauritania OR Niger OR Nigeria OR Senegal OR Côte d’Ivoire OR “Sierra Leone” OR Togo OR Angola OR Cameroon OR “Central African Republic” OR Chad OR Congo OR “Republic of the Congo” OR “Democratic Republic of the Congo” OR “DR Congo” OR “Equatorial Guinea” OR Gabon OR “São Tomé and Príncipe”)
ProQuest	(“men who have sex with men” OR MSM OR “homosexual men” OR homosexual* OR gay OR bisexual* OR “sexual minority men” OR “sexual minority groups”) AND (“condom use” OR condoms OR “male condom” OR PrEP OR “pre-exposure prophylaxis” OR “HIV prevention” OR “HIV risk reduction” OR “sexual risk reduction” OR “safe sex”) AND (HIV OR AIDS OR “human immunodeficiency virus” OR “acquired immunodeficiency syndrome” OR “HIV infection”) AND (“West Africa” OR “Central Africa” OR Benin OR Burkina Faso OR “Cape Verde” OR “Cabo Verde” OR Gambia OR Ghana OR Guinea OR “Guinea-Bissau” OR Liberia OR Mali OR Mauritania OR Niger OR Nigeria OR Senegal OR Côte d’Ivoire OR “Sierra Leone” OR Togo OR Angola OR Cameroon OR “Central African Republic” OR Chad OR Congo OR “Republic of the Congo” OR “Democratic Republic of the Congo” OR “DR Congo” OR “Equatorial Guinea” OR Gabon OR “São Tomé and Príncipe”)”
PsycINFO	(“men who have sex with men” OR MSM OR “homosexual men” OR homosexual* OR gay OR bisexual* OR “sexual minority men” OR “sexual minority groups”) AND (“condom use” OR condoms OR “male condom” OR PrEP OR “pre-exposure prophylaxis” OR “HIV prevention” OR “HIV risk reduction” OR “sexual risk reduction” OR “safe sex”) AND (HIV OR AIDS OR “human immunodeficiency virus” OR “acquired immunodeficiency syndrome” OR “HIV infection”) AND (“West Africa” OR “Central Africa” OR Benin OR Burkina Faso OR “Cape Verde” OR “Cabo Verde” OR Gambia OR Ghana OR Guinea OR “Guinea-Bissau” OR Liberia OR Mali OR Mauritania OR Niger OR Nigeria OR Senegal OR Côte d’Ivoire OR “Sierra Leone” OR Togo OR Angola OR Cameroon OR “Central African Republic” OR Chad OR Congo OR “Republic of the Congo” OR “Democratic Republic of the Congo” OR “DR Congo” OR “Equatorial Guinea” OR Gabon OR “São Tomé and Príncipe”)
OpenAIRE	MSM and PrEP and condom and Africa

### Critical Appraisal

Critical appraisal was not conducted for this study, as this is not a mandatory requirement for a scoping review [20].

### Data Extraction and Analysis

We mapped the scope of existing research, described study characteristics, and synthesised the findings to produce a descriptive overview aligned with the objectives of the scoping review [24]. Data from the selected studies were extracted and summarised into a standardised table using a data charting approach, followed by a narrative synthesis to provide a structured overview of the evidence [24]. One author extracted the data from eligible articles included in the scoping review using a customised data extraction template designed by the research team to summarise key characteristics from the eligible studies, such as author(s) and publication year, study title, country of origin, study design, study population and sample size, key findings and relevance to the scoping review questions. Two other authors independently reviewed the extracted data (data summary table) to ensure the appropriateness of information and relevance to the scoping review. Discrepancies identified during this process were discussed collaboratively among the reviewers until a joint resolution was achieved.

Following the data extraction process, in line with the review objectives, we conducted an inductive thematic analysis [22]. We identified and interpreted patterns in the data and described the key themes [20, 22]. Finally, we presented the findings as a narrative synthesis while outlining the studies contributing to the synthesis. The initial synthesis was conducted by the first author, and two other authors independently verified the accuracy and completeness of the information. Any discrepancies identified during this process were discussed collaboratively among the reviewers until a joint resolution was achieved.

## Results

### Selection Process for the Sources of Evidence

Studies were selected through a structured screening process guided by clearly defined inclusion criteria. Titles were reviewed to identify studies explicitly referencing PrEP and/or condom use among MSM in Western and Central African countries. The team iteratively refined the search strategy during the abstract review process until the final search strategy was established. The first author conducted the initial search and reviewed the abstracts. Two team members independently assessed the abstracts for relevance, and subsequently, reviewed the full-length articles for accuracy and completeness. A third team member acted as a tiebreaker in cases of disagreement. Studies that reported on PrEP uptake, condom use, or both among MSM were included in the final evaluation.

Overall, 713 articles were identified during the search process. Of these, 701 were retrieved through database searches across CINAHL Ultimate (EBSCO), ProQuest, Scopus, PsycINFO, PubMed, and OpenAIRE, while an additional 12 studies were identified through manual screening of reference lists and websites. After removing 139 duplicates, 519 articles remained for screening based on titles and abstracts. Following this initial screening, 55 articles were selected for full-text review, of which 33 were subsequently excluded for various reasons.

**Fig. 1:**
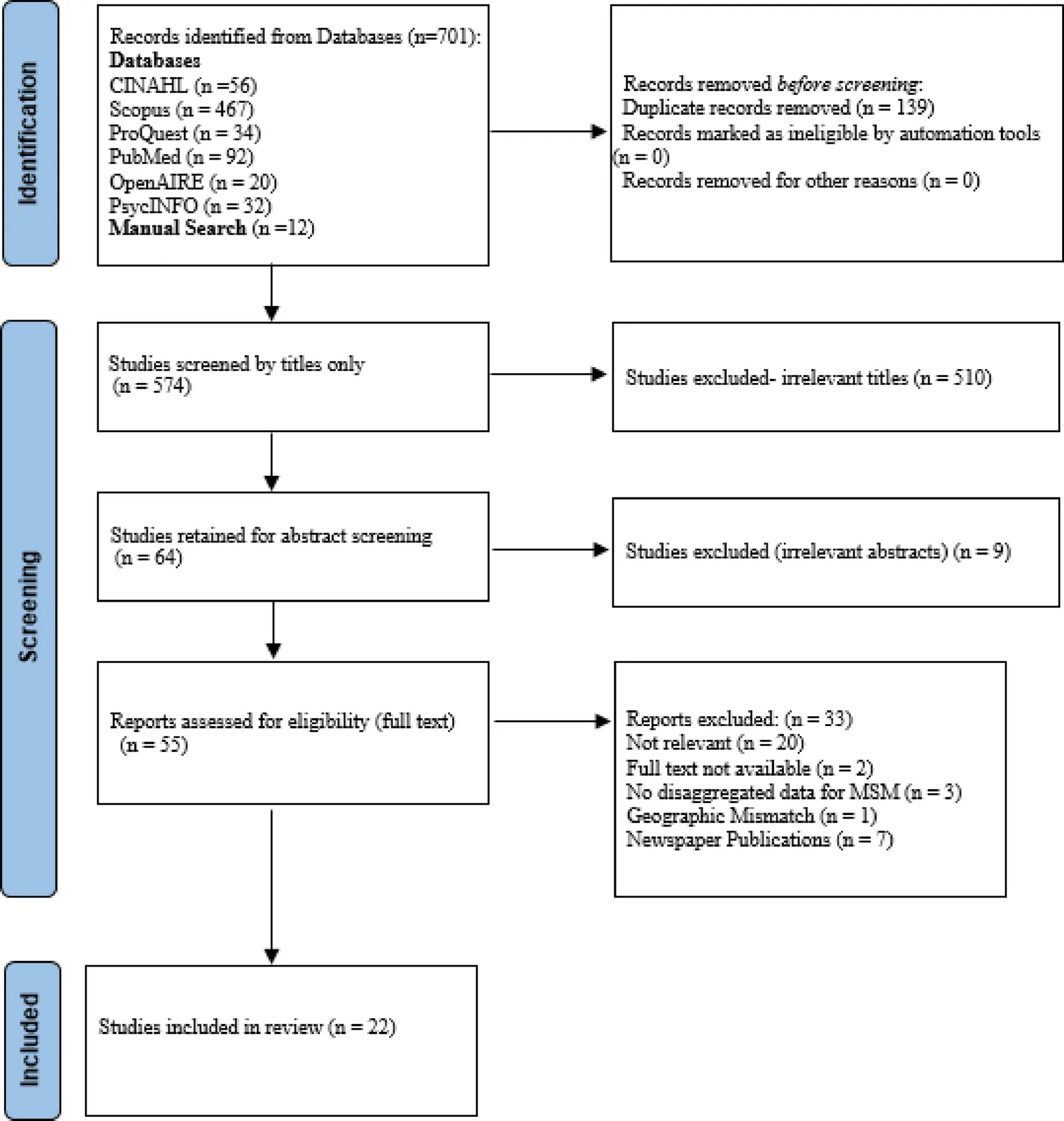
PRISMA flow diagram. Adapted from: Page MJ, McKenzie JE, Bossuyt PM, Boutron I, Hoffmann TC, Mulrow CD, et al. The PRISMA 2020 statement: an updated guideline for reporting systematic reviews. BMJ 2021;372:n71. doi: https://doi.org/10.1136/bmj.71.

Twenty studies (20) were excluded due to a lack of relevance. Two (2) studies were excluded because full-text access was unavailable. Three (3) studies were excluded because they did not provide disaggregated data for the population of interest, and one (1) was excluded due to geographic mismatch, as it was conducted in SSA but did not include countries from the West or Central African regions. Additionally, seven (7) articles were excluded because they were newspaper publications rather than peer-reviewed research. Consequently, 22 studies met the inclusion criteria and were included in the final review. The search results were exported into EndNote software to facilitate accurate citation and efficient source management [23]. Articles that met the inclusion criteria were selected, and the screening process is presented in the PRISMA-ScR flow diagram (Fig. 1).

### Overview of Study Characteristics

Of the 22 studies included, nine studies (40.9%) employed a cohort study design, ten (45.5%) were cross-sectional studies, and three (13.6%) used qualitative methods. In terms of publication dates, 13 studies (59.1%) were published between 2017 and 2020, while the remaining nine studies (40.9%) were published between 2021 and 2025. Geographically, the majority of the studies (21 studies; 95.5%), were conducted in various countries across West Africa; only one study (4.5%) was conducted in the Central African region. Detailed study characteristics are presented in Table 3.

**Table 3:** Characteristics of included studies (*n* = 22)

Study characteristics		*n* (%)
Publication year	2017–2020 2021–2025	13 (59.1) 9 (40.9)
Country of study	Ghana Nigeria Togo Cameroon Benin Mali Mali, Côte d’Ivoire, Burkina Faso, Togo	3 (13.7) 6 (27.3) 2 (9.1) 1 (4.6) 2 (9.1) 1 (4.5) 7 (31.8)
Study population	Gay, bisexual, and other men who have sex with men (GBMSM) MSM MSM and Transgender women (TGW) FSW and MSM MSM, FSW and People who inject drugs (PWID)	2 (9.1) 17 (77.3) 1 (4.6) 1 (4.5) 1 (4.5)
Study design	Qualitative Cohort study Cross-sectional study	3 (13.6) 9 (40.9) 10 (45.5)

### Data Charting Summary

A standardised data charting summary is presented in Table 4, comprising collated information and summarised key characteristics from the eligible studies. This includes details such as author(s) and publication year, study title, country of origin, study design, study population and sample size, key findings and relevance to the scoping review questions. This data charting summary also constitutes the data extraction table, which is presented in Table 4 and appears after the PRISMA-ScR Checklist.

Our review identified three major themes across the included studies: (1) patterns and trends in PrEP adoption and associated factors; (2) condom use patterns and practices; and (3) combined PrEP and condom use with emerging risk compensation concerns.

### Patterns and Trends in PrEP Adoption and Associated Factors

Overall, five studies reported on patterns of PrEP uptake among MSM in WCA [25–29]. Patterns of uptake, defined as ever using PrEP, varied across countries within the region. Low PrEP uptake was observed in Nigeria (29.7% among GBMSM) [29], Ghana (17.8% among MSM) and Cameroon (7% among MSM) [25, 26]. In contrast, a multi-country study in Mali, Côte d’Ivoire, Burkina Faso, and Togo reported a high PrEP uptake of 70% among MSM who engaged in anal intercourse [28]. This high uptake of PrEP was reported among those who engaged in condomless anal sex or reported receptive anal intercourse. In Ghana, geographical disparities were observed; uptake was lower in the Upper West and Eastern regions compared to Volta, Brong Ahafo, Ashanti, Western, and Greater Accra regions [25]. These differences were attributed to structural, relational, and behavioural factors [25]. Notably, age was not identified as a significant determinant of PrEP uptake in this context [25].

### Condom Use Patterns and Practices

Most studies revealed country-specific trends and variability in condom use patterns among MSM [26–28, 30–44]. Consistent condom use (CCU), defined as correct and consistent condom use during every sexual encounter during a predefined recall period, was low to moderate across the West and Central African region, with variation based on country and study population. In Cameroon, Ghana, and the Benin Republic, Integrated Biological and Behavioural Surveys (IBBSs) reported CCU at 41%, 48.2%, and 34%, respectively [26, 27, 30]. Similarly, in Mali and Togo, about 20–30% of MSM did not use condoms consistently during anal intercourse [31, 32]. Across Mali, Côte d’Ivoire, Burkina Faso, and Togo in a multi-country study, CCU during anal sex in the six months preceding study enrolment remained suboptimal, with 54% of participants reporting inconsistent use [33]. In Nigeria, MSM were significantly less likely to use condoms compared to other key populations (adjusted odds ratio of 0.17 (95% CI: 0.05–0.67; *p* = 0.01) [35]. While 90.3% of MSM reported CCU during anal sex, 20% still engaged in condomless sex [35]. In Togo, a marked improvement was observed in MSM between 2011 and 2015, with CCU increasing from 21.6% to 68.5% for insertive anal sex and from 18.1% to 71.9% for receptive anal sex [31].

Partners’ HIV status may influence condom use. Evidence from Mali, Côte d’Ivoire, Burkina Faso, and Togo indicated inconsistent condom use over the six months preceding enrolment, particularly during receptive anal sex with male partners of unknown HIV status [38]. In a separate study, in the same region, it was established that 20% of sexual activities among MSM were protected by condom use alone [42]. Among HIV-positive MSM, condom use patterns varied; some individuals increased condom use following diagnosis, driven by a heightened sense of responsibility and interactions with healthcare providers [44]. Nevertheless, inconsistencies remained in condom use behaviour; while 67.5% of HIV-positive MSM reported condom use during their most recent sexual encounter, about one-third still engaged in condomless anal sex [39].

Relationship dynamics and the type of sexual partner further influenced preventive behaviours among MSM [30, 32, 34, 36, 43, 44]. In Nigeria, condom use during transactional sex increased from 35% in 2007 to 79% in 2014, while use during non-transactional sexual encounters remained relatively stagnant at approximately 50% [34]. This suggests trust-based risk where MSM accurately perceive professional risk, but underestimate risk within trusted social or romantic circles. In Togo, while low rates of condom use were observed, older age, higher income, and improved condom accessibility positively influenced CCU, with the highest rates reported during anal intercourse with casual male partners [36]. MSM were significantly more likely to use condoms with male or casual partners than with female or long-term partners. For instance, in Bamako (Mali), condom use was 76% with male partners versus 43% with female partners [32]. In Ghana, CCU was higher in high-risk contexts, such as during casual sex or post-STI diagnosis, but declined in long-term, emotionally intimate relationships due to trust and familiarity [44]. Similar trends occurred in Akwa Ibom, Nigeria, where only 2% of MSM used condoms consistently with all partners, while 50% preferred condomless sex with male partners and 34% with both male and female partners [43]. Additionally, in Ghana, consistent use during anal sex was 48.2% with male partners and even lower (29.7%) with female partners [30]. Finally, condom use varied by sexual role. In Mali, Côte d’Ivoire, Burkina Faso, and Togo, inconsistent use was reported by 30.3% of MSM during insertive anal sex and 33.7% during receptive anal sex over a six-month period [40].

### Combined PrEP and Condom Use With Emerging Risk Compensation Concerns

Dual prevention strategies have the potential to reduce HIV transmission. However, the data on their combined use remains sparse. In the only study [42] addressing the combined use of condoms and PrEP, correct adherence to PrEP and condom use was reported in 30% of sexual encounters among MSM, while incorrect adherence to both occurred in 11%.

Reduced risk perceptions following PrEP initiation, a phenomenon often termed risk compensation, were identified with PrEP and condom use, and sexual practices in this review. Such behavioural shifts were evidenced in one study [28], with two other studies [45, 46] pointing to similar behaviours. Specifically, a multi-country study across Mali, Côte d’Ivoire, Burkina Faso, and Togo revealed that higher utilisation of PrEP was associated with condomless anal sex and increased sexual activity with long-term partners [28]. These behavioural shifts included PrEP uptake and inconsistent condom use [33, 46], anticipated increased sexual activity with a high number of sexual partners [46] and uninhibited condomless sex [45].

## Discussion

This scoping review synthesised existing research on the adoption of PrEP and condom use among MSM in WCA. The findings offer key critical insights into the HIV prevention landscape for MSM in this region, particularly regarding the uptake of PrEP and condom use practices.

Although PrEP has emerged as a highly effective biomedical approach for HIV prevention, its uptake and adherence remain uneven across different populations and regions, particularly among MSM [47–49]. While some reviewed studies, such as in Burkina Faso, Côte d’Ivoire, Mali, and Togo, revealed high PrEP uptake, others in Ghana, Nigeria, and Cameroon showed low PrEP uptake, with further variability observed within regions in Ghana. These challenges persist and underscore the need for initiatives targeted at increasing PrEP uptake in WCA as global access to PrEP expands. By 2023, more than 3.5 million individuals had received PrEP at least once; approximately 2.6 million of them residing in Africa, accounting for more than 75% of the global total [1]. Generally, low CCU is a prevailing concern among MSM except in Togo.

While some studies indicated high levels of awareness and interest in using PrEP among MSM, actual usage remains limited in the West and Central African region [25, 26, 29]. This aligns with broader patterns observed in the United States [50–53], China [54], Canada [55], and throughout SSA, where numerous challenges hinder the transition from awareness to consistent use [56]. The generally low PrEP adoption rate among MSM in WCA is consistent with the global estimate of 16.0% [57]. Our findings regarding geographic disparities in PrEP uptake in WCA underscore the necessity for innovative and context-sensitive delivery strategies.

Reduced condom use despite increasing HIV prevalence among MSM is a significant concern, making PrEP uptake an essential prevention strategy [58]. Variations in condom use across partner types and relationship contexts reflect deeper relational and psychological factors that shape sexual decision-making [59]. In alignment with previous studies, our review indicates that MSM are more likely to use condoms in casual or transactional encounters than in long-term or emotionally close relationships, where trust and emotional intimacy may lower perceived risk and reduce condom use motivation [32, 34, 44, 60]. This study further establishes that condom use is often discontinued in stable relationships, revealing a “protection gap” that necessitates complementary biomedical interventions like PrEP. These dynamics suggest that prevention efforts need to adopt initiatives that focus on relationship dynamics, such as couple-based counselling [5] and communication skills training for HIV program counsellors to foster safer sexual practices among MSM [61].

Our review further identifies dual prevention, PrEP combined with condom use, as an underutilised HIV prevention strategy. Condom use patterns were influenced by biomedical interventions, reflecting risk compensation, defined as increased risk-taking linked to perceived protection from adverse consequences [62, 63]. As revealed in our review, PrEP initiation can result in risky behavioural changes such as decreased condom use [64–68] or increased sexual activity/partners among MSM [69–73]. However, evidence from Kenya [74] and India [75] has reported increased condom use among MSM on PrEP. This indicates that PrEP does not necessarily diminish condom use when delivered through well-supported, routine services with reliable condom access. This is consistent with a study in Nigeria where no form of risk compensation about condom use practice was observed among MSM who use PrEP [76].

Despite the quantitative evidence regarding patterns of PrEP uptake and condom use, this review highlights gaps that limit a deeper understanding of prevention behaviours in WCA. There is limited insight into the lived experiences of MSM and the adoption of HIV prevention measures due to the paucity of qualitative studies. Although this review reported patterns of PrEP uptake, low condom use, and risk compensation, existing literature sheds little light on how MSM navigate the transition from condoms to PrEP or how relationship dynamics and partner expectations shape these decisions. There is a pressing need for studies that foreground MSM voices regarding how PrEP awareness initiatives should be structured and which community-led approaches would strengthen its uptake in ways that best reflect the distinct cultural and contextual realities of the region.

This scoping review recognises several inherent limitations. We included only studies published in English, which may have led to the exclusion of pertinent evidence from non-English-speaking (Francophone or Lusophone) countries in the WCA. Furthermore, only one study from Central Africa met the criteria for inclusion in the review. Thus, our conclusions may not be generalisable to the entire Central African region. Lastly, the documented outcomes may not fully represent current adoption levels due to the scarcity of literature from certain West and Central African countries.

The strengths of this review lie in the use of a well-defined search strategy across multiple databases and a systematic approach based on JBI methodological guidelines. Although only three of the twenty-two included studies used qualitative methods, this limited evidence base actually strengthens the review’s contribution by exposing a clear methodological gap in research that could illuminate how MSM understand and navigate PrEP adoption alongside declining condom use. By highlighting how little is known about these lived experiences, this review underscores the need for deeper, more nuanced inquiry in WCA.

## Conclusion

This review maps the current evidence on the adoption of PrEP and condom use among MSM in WCA. The findings reveal uneven PrEP uptake, persistent inconsistencies in condom use, and emerging behavioural shifts associated with PrEP initiation, all of which are shaped by complex structural and relational factors. These insights emphasise the need for context-sensitive, community-aligned prevention strategies and highlight critical areas where qualitative research is urgently required to deepen the understanding of MSM’s lived experiences and PrEP utilisation. Taken together, this review provides a foundation for future research and tailored interventions aimed at strengthening HIV prevention efforts for MSM across the region.

**Table 4:** Data extraction table- Summary of data from eligible studies

Authors	Study Title	Country	Study design	Population/sample	Key findings	Relevance to review question
Cameroon Ministry of Public Health (2017)	2016 Integrated Biological and Behavioural Survey (IBBS) Report among Key Populations in Cameroon: Female sex workers (FSW) and men who have sex with men	Cameroon	Cross-sectional	2255 FSW and 1323 MSM	The study revealed that 81.7% of MSM reported using a condom during their last sexual encounter. Overall, 7% of MSM reported having used ARVs for HIV prevention (PrEP). The findings showed a reliance on condoms as the main prevention method, with little variation in strategies. The absence of PrEP suggests a gap in understanding broader prevention behaviours.	The study highlights the pattern of PrEP uptake and CCU among key populations.
Ghana AIDS Commission (2017)	Mapping and population size estimation (MPSE) and integrated bio-behavioural surveillance survey (IBBSS) amongst men who have sex with men (MSM) in Ghana (Round II)	Ghana	Cross-sectional	4,095 MSM	The study revealed that a significant majority of MSM (over 80%) were aware of and had used condoms at some point. Despite this high awareness, consistent condom use (CCU) was notably low, especially with non-commercial and regular partners. During the survey, PrEP was not widely available in Ghana, resulting in a lack of documented behaviours or patterns. The study identifies gaps in CCU and the lack of PrEP in HIV prevention strategies	The study highlights the pattern of CCU among MSM.
Teclessou, et al. (2017)	Evolution of HIV prevalence and behavioural factors among MSM in Togo	Togo	Serial cross-sectional surveys	496 MSM	In the 30 days leading up to the survey, 68.5% of respondents indicated CCU during insertive anal intercourse, while 71.9% utilised condoms during passive anal sexual activity.	Presents a pattern of condom use among MSM
Lahuerta et al. (2018)	HIV Prevalence and Related Risk Factors in Men Who Have Sex with Men in Bamako, Mali: Findings from a Bio-behavioural Survey Using Respondent-Driven Sampling	Mali	Cross-sectional study	552 MSM	Condom utilisation during anal sexual activity with male partners was stated by 76% of MSM	Illustrates condom use trend in MSM.
Coulaud et al. (2018)	Interest in HIV pre-exposure prophylaxis in men who have sex with men in West Africa (CohMSM ANRS 12324 – Expertise France)	Mali, Côte d’Ivoire, Burkina Faso, Togo	Cohort study	564 MSM	Of all respondents, 54% reported inconsistent condom use during anal sex in the past six months, while 87% expressed interest in PrEP utilisation	Illustrates geographic patterns in PrEP interest and condom usage among MSM.
Ogunbajo, A., et al. (2019)	Awareness, willingness to use, and history of HIV PrEP among MSM in Nigeria	Nigeria	Cross-sectional survey	400 Gay, bisexual, and MSM (GBMSM)	PrEP use was found to be low, with 29.7% reporting prior utilisation in spite of high awareness and willingness. Out of all respondents, 46.4% lacked prior PrEP awareness, but 80.1% were ready to utilise it following education about its advantages in preventing HIV.	Gives vital insight into PrEP uptake among GBMSM
Eluwa et al. (2019)	Rising HIV prevalence among men who have sex with men in Nigeria: A trend analysis	Nigeria	Cross-sectional study	6035 MSM	The study reports growing HIV prevalence in MSM with reduced and irregular condom use. Growing condom utilisation was reported during transactional sex engagement over time, from 35% in 2007 to 79% in 2014. In contrast, condom use in non-transactional sexual encounters continued to lag behind, stabilising at about 50% throughout that timeframe.	Highlights investigation into condom use patterns.
Ruiseñor-Escudero et al. (2019)	CCU Among Men Who Have Sex with Men in Lomé and Kara, Togo	Togo	Cross-sectional study	683 MSM	This study found low rates of reported condom use. MSM in rural areas (Kara) are likely to use condoms inconsistently as compared to those living in urban areas (Lomé). In Lomé, MSM reported more CCU during anal intercourse with male partners over the past month during the study, with the highest rates observed when engaging with casual partners.	Highlights regional variation in condom use and practices among MSM
Ochonye et al. (2019)	Sexual practices, sexual behaviour and HIV risk profile of key populations in Nigeria	Nigeria	Cross-sectional	145 MSM, 188 FSW, 155 PWID	90.3% of MSM reported CCU during anal sex, but 20% still engaged in condomless sex when partners refused a condom in the last 12 months preceding the study	Offers behavioural patterns and condom usage trends among MSM in Nigeria.
Ahouada, C., et al. (2020)	Acceptability of PrEP among MSM in Benin: A qualitative study	Benin	Qualitative study	36 MSM	A little more than half of the respondents acknowledged the possibility of PrEP leading to reduced condom use or its complete discontinuation.	Points out concerns about risk compensation following PrEP use.
Afolaranmi et al. (2020)	Sexual Behaviours of HIV-Infected Men who Have Sex with Men in Jos, Plateau State, North Central Nigeria	Nigeria	Cross-sectional survey	114 MSM	The findings reveal that 67.5% of respondents utilised condoms in their last same sex engagement, while condoms were used by 61% of MSM who had bisexual sex encounters in their last sexual activities	Examines patterns of condom use among HIV infected MSM
Coulaud et al. (2020a)	Combined Sexual Behavioral Profiles in HIV-Seronegative Men Who have Sex with Men in West Africa (CohMSM ANRS 12324-Expertise France)	Mali, Côte d’Ivoire, Burkina Faso, Togo	Observational cohort study	520 MSM	The study reported that nearly half (48.3%) of all sexual encounters involve inconsistent condom use, and nearly 52% of MSM demonstrated inconsistent condom use patterns, with the proportions falling between those at high risk during receptive (22%) and insertive (30%) sexual practices.	Identifies condom use patterns and practices
Coulaud et al. (2020b)	Changes in risky sexual behaviours among West African MSM enrolled in a quarterly HIV testing and counselling prevention programme (CohMSM ANRS 12324 - Expertise France)	Mali, Côte d’Ivoire, Burkina Faso, Togo	Longitudinal cohort study	621 MSM	During the study period, respondents reported inconsistent condom use over the preceding six months, during receptive anal sex with male partners of unknown HIV status. However, engagement in risky sexual practices, such as inconsistent condom use, declined over time, associated with regular counselling and routine HIV testing.	The study documents condom use behaviour over time
Dah et al. (2021)	Adherence to quarterly HIV prevention services and its impact on HIV incidence in men who have sex with men in West Africa (CohMSM ANRS 12324 - Expertise France)	Burkina Faso, Côte d’Ivoire, Mali, Togo	Cohort study (CohMSM)	618 MSM	A large number of respondents reported irregular condom use during intercourse. Particularly, 30.3% of respondents stated irregular condom utilisation during insertive anal sexual activity, whereas 33.7% of respondents indicated the same during receptive anal sex. At the time of the study, PrEP was not accessible to the participants.	Shows patterns of condom utilisation in MSM.
Olawore et al. (2021)	Individual and partnership characteristics associated with consistent CCU in a cohort of cisgender men who have sex with men and transgender women in Nigeria	Nigeria	Cohort study	2591 Cisgender MSM and transgender women (TGW)	Variations in CCU were observed based on the type of relationship. CCU was greater in relationships with well-educated partners, partners’ encouragement of condom use, casual relationships, an expansive MSM network, and where HIV was talked about once weekly. Lower condom use was associated with committed relationships, while partners with unknown or positive HIV status, and/or where a partner was in a marriage with a woman, were associated with reduced CCU.	Highlights differences in condom use patterns across relationship types in MSM
Eubanks et al. (2022a)	Socio-behavioural correlates of pre-exposure prophylaxis use and correct adherence in men who have sex with men in West Africa	Mali, Côte d’Ivoire, Burkina Faso, Togo	Cross-sectional analysis from CohMSM	632 MSM	During the study, 70% of recent anal sex involved PrEP use. Higher PrEP utilisation was noted with condomless anal sex (CAS) and increased sexual intercourse with long-term partners. A decline in the use of condoms following PrEP initiation was also noted	Shows behavioural patterns linked to PrEP and shifts in condom use following PrEP initiation.
Eubanks et al. (2022b)	Rate and Predictors of Ineffective HIV Protection in African Men Who Have Sex with Men Taking Pre-Exposure Prophylaxis	Burkina Faso, Côte d’Ivoire, Mali, Togo	Cohort study (CohMSM)	632 MSM	Regarding HIV prevention, 30% of sexual encounters were protected by both correct PrEP and condom use, 22% relied solely on correct PrEP adherence, 20% were protected by only condom use and 11% involved incorrect PrEP adherence alongside condom use	Showcases condom and PrEP use patterns in MSM
Abu-Ba’are et al., 2023	Awareness and Willingness to use Condoms and Preexposure Prophylaxis among Gay, Bisexual, and Other Cisgendered Men who Have sex with men (GBMSM) in Slum Communities in Ghana. BSGH-004	Ghana	Qualitative (in-depth interviews)	12 GBMSM	Condom utilisation in GBMSM is shaped by trust and long-term stability, often leading to reduced consistency even among individuals who were aware of HIV risks. Condom usage is often intermittent due to partner negotiation power, and it is often abandoned in long-term relationships.	Highlights the condom use practice among GBMSM
Reyniers et al. (2023)	Strategies to improve PrEP uptake among West African men who have sex with men: a multi-country qualitative study	Mali, Côte d’Ivoire, Burkina Faso, Togo	Qualitative study	97 MSM	Participants generally held favourable views on PrEP, thinking it could alleviate challenges associated with condom use.	Underscores the potential behavioural changes following PrEP initiation, including reduced condom use.
Izuchukwu et al. (2023)	Risk factors of HIV/AIDS among men who have sex with men in Akwa Ibom State, Nigeria	Nigeria	Cross-sectional survey	400 MSM	The study indicates low condom use. Half of the participants liked unprotected sex with male partners, while 34% leaned toward sex without condoms with both male and female partners, and 14% only stated condomless sex with female partners. Regarding PrEP awareness, variations were observed; 34.2% were informed, 31% knew about it but didn’t use it, and 34.8% had no previous PrEP knowledge.	Shows PrEP and condom use patterns
Diabaté et al. (2023)	Pre-exposure prophylaxis in real life: experience from a prospective, observational and demonstration project among men who have sex with men in Benin, West Africa	Benin	Prospective observational and demonstration project	204 MSM	Low CCU was documented, with about 34% initially, rising to 37% by month-6 and levelling off at 36% by month-12.	Spotlights condom utilisation patterns in Benin MSM.
Apreku et al. (2025)	Awareness, willingness, and uptake of PrEP among MSM in Ghana	Ghana	Cross-sectional study	2627 MSM	Despite moderate awareness and high willingness, low PrEP use was reported, with only 17.8% of participants having ever utilised PrEP.	Highlights the PrEP uptake pattern
